# Blood and faecal biomarkers to assess dietary energy, protein and amino acid efficiency of utilization by growing and finishing pigs

**DOI:** 10.1186/s40813-022-00273-y

**Published:** 2022-07-04

**Authors:** Jordi Camp Montoro, David Solà-Oriol, Ramon Muns, Josep Gasa, Núria Llanes, Edgar Garcia Manzanilla

**Affiliations:** 1 Pig Development Department, Animal and Grassland Research and Innovation Centre, Teagasc, Moorepark, Fermoy, Co. Cork, P61 C996 Ireland; 2 Department of Animal and Food Sciences, Animal Nutrition and Welfare Service, Universitat Autònoma de Barcelona, 08193 Bellaterra, Spain; 3 Agri-Food and Biosciences Institute, Large Park, HillsboroughBT 26 6DR, Co Down, Northern Ireland UK; 4 Cooperativa d’Ivars d’Urgell SCCL, 25260 Ivars d’Urgell, Lleida, Spain; 5 UCD Veterinary Sciences Centre, University College Dublin, Belfield, Dublin 4, D04 V1W8 Ireland

**Keywords:** Faeces, Growth, Lysine, Metabolism, Nutrition, Requirements, Swine

## Abstract

**Background:**

Diet evaluation and optimization is a slow and expensive process and it is not possible to do it at a farm level. This study aimed to use the blood serum metabolite (BSM) and faecal volatile fatty acid (VFA) profiles as potential biomarkers to identify changes in protein, amino acid and energy dietary content in growing and finishing pig diets at farm level.

**Results:**

Two studies were conducted. The first study (S1) included 20 pens of 11 pigs (87.0 ± 4.10 kg; 18 weeks old) assigned to 5 diets: control (C1), high or low crude protein (HP1 and LP1, respectively), and high or low net energy (HE1 and LE1, respectively). The second study (S2) included 28 pens of 11 pigs (41.3 ± 2.60 kg; 12 weeks old) assigned to 7 diets: control (C2), high or low crude protein (HP2 and LP2, respectively), high or low amino acid (HA2 and LA2, respectively), and high or low net energy (HE2 and LE2, respectively). Pigs were followed for 10 (S1) and 20 (S2) days, and blood and faecal samples were collected at 20 (S1) and 14 (S2) weeks of age. Data were analysed using general linear models and receiver operating characteristic curve analysis. Urea nitrogen showed the best results as a biomarker. Urea nitrogen was higher in pigs fed high protein diets, HP1 (13.6 ± 0.95 mg/dL) and HP2 (11.6 ± 0.61), compared to those fed low protein diets, LP1 (6.0 ± 0.95) and LP2 (5.2 ± 0.61; *P* < 0.001), showing good discrimination ability (Area under the curve (AUC) = 98.4 and 100%, respectively). These differences were not observed between diets LA2 (6.5 ± 0.61) and HA2 (8.7 ± 0.61; *P* > 0.05; AUC = 71.9%), which were formulated based on the ideal protein profile but with no excess of protein. Creatinine, triglycerides, branched-chain fatty acids, albumin, propionic acid, and cholesterol showed differences between at least 2 diets but only in one of the studies.

**Conclusions:**

Urea nitrogen showed high accuracy to detect excess of crude protein in growing and finishing pig diets. Other biomarkers like BCFA showed promising results and need to be further studied.

## Background

Pig diets are formulated with the goal of optimising growth, health, and welfare of the animals by meeting their nutritional requirements while avoiding the excess of nutrients, especially nitrogen and phosphorus, that result in environmental pollution. However, the actual nutritional value of a diet for a particular pig farm is affected by different factors such as quality of ingredients [1], feed form and delivery method [2], variability of body weight **(BW)** [3, 4], farm management [5, 6], or health status of the farm [7, 8], among others. With so many factors affecting nutritional value of feed, suboptimal diets are not rare and can result in extra cost for the farmers, potential health and welfare problems for the pigs, and environmental contamination.

The use of low protein diets, including more synthetic amino acids **(AA**s**)**, is one of the most effective measures to reduce the incidence of diarrhoea in pigs [9] and to reduce ammonia emissions in pig farms [10]. However, the optimum levels of AAs and the balance between AAs and energy can vary depending on the particularities of each farm, and the same low protein diet can be adequate or not depending on the farm. Thus, methods to optimise diets at farm level are needed. Classical methods for feed assessment, such as digestibility trials, are expensive, time consuming, and are not suited for use in commercial farms [11]. Fast methods to optimise diets at individual farm level would be of interest to improve production efficiency, animal health and welfare, and to reduce the environmental footprint and production costs [12–14].

Blood biochemistry is a fast analysis method used regularly in clinical practice for many animal species and it can provide parameters directly related to energy and protein metabolism. Dietary changes affect blood metabolites such as total protein [15–17], albumin [16–18], serum urea nitrogen **(SUN)** [19–21] and creatinine [21] for the protein metabolism, and glucose [15–17], triglycerides [18, 22] and cholesterol [17, 18] for the energy metabolism.

Faecal samples are easy to collect in pig farms and fermentation components present in faeces, like volatile fatty acids **(VFA**s**)** can be easily measured and are directly affected by the composition of the diet and the metabolism of nutrients by the animal [23–25]. Carbohydrates are catabolized to short-chain fatty acids such as acetic, propionic or butyric acid, while protein results in a higher concentration of branched-chain fatty acids **(BCFA)** produced from the deamination of branched-chain amino acids such as leucine, isoleucine, and valine [25]. Therefore, high proportions of BCFA measured in faeces could indicate an excess of protein reaching distal parts of the intestine and being inefficiently used by pigs.

Blood serum metabolites and faecal VFA profiles could be potential biomarkers to identify diets that are suboptimal at individual farm level. Therefore, we hypothesise that blood metabolites and faecal VFA profiles are biomarkers sensible enough to reflect changes in dietary energy, protein, and AA for growing and finishing pigs at farm level. The objective of the present study was to evaluate the use of the blood serum metabolite and faecal VFA profiles as potential biomarkers to identify differences in the levels of dietary net energy **(NE**), crude protein **(CP)**, and AA within normal ranges for growing and finishing pigs at farm level.

## Methods

### Animals, diets, and experimental design

Two studies were conducted in the Teagasc Pig Research Facility in Fermoy, Co. Cork, Ireland. Both studies received ethical approval from the Teagasc Animal Ethics Committee (TAEC 244/2019). In both studies, pigs were housed in pens with fully slatted concrete floor (2.4 × 4.2 m) containing a single wet-dry feeder (330 mm [Width] × 370 mm [Depth] × 1000 mm [Height]; MA37, Verba, Netherlands) and one supplementary nipple drinker. Water and pelleted feed were provided ad libitum. Temperature was controlled by a mechanical ventilation system with fan speed and air inlet area regulated by a climate controller. Pens were enriched with a larch wood post.

The first study (**S1**) was conducted in finishing pigs from 18 to 20 weeks of age (87.0 ± 4.10 to 100.3 ± 4.40 kg of BW). A total of 220 Danish Duroc × (Large White × Landrace) growing pigs born within one week were moved to the grower-finisher stage at 11 weeks of age and housed in balanced mixed sex pens. Pigs were fed a single soybean meal-maize-wheat based grower-finisher diet (9.67 MJ/kg of NE, 16.2% of CP, and 0.92% of standard ileal digestive **(SID)** lysine **(Lys)**) from 11 to 18 weeks of age before the study started. The study started at 18 weeks of age when pigs were weighed per pen (n = 20; 11 pigs/pen) and assigned per pen based on BW to five different dietary treatments following a complete randomized design. Pigs were followed for 10 days. Diets were formulated to obtain a control diet (**C1**; 10.03 MJ/kg of NE, 16.0% of CP, and 0.95% of SID Lys) which met or exceed the minimum nutrient requirements [26], and 4 modifications of this diet: low crude protein **(LP1)** by reducing CP to 13.2% and AAs to 0.75% of SID Lys; high crude protein **(HP1)** by increasing CP to 18.8% and AAs to 1.15% of SID Lys; low net energy **(LE1)** by reducing NE to 9.61 MJ/kg; and high net energy **(HE1)** by increasing NE to 10.45 MJ/kg. Ingredient, calculated, and analysed nutrient diet composition is shown in Table 1. Pigs went back to the common management of the Teagasc Pig Research Facility after the 10-day trial period.

**Table 1:** Ingredient, calculated, and analysed nutrient composition on an as-fed basis of the five dietary treatments in study 1^a^

	Diets^b^				
	C1	LP1	HP1	LE1	HE1
Ingredients, g/kg					
Wheat	350.0	350.0	350.0	330.0	306.2
Barley	282.5	345.0	0.0	310.5	200.0
Maize	150.0	150.0	286.6	100.0	275.5
Soybean meal 47.5	172.4	95.7	254.1	175.1	172.4
Soybean hulls	14.2	29.7	63.9	58.3	0.0
Vegetable oil	5.0	5.0	17.6	0.0	21.5
Calcium carbonate	12.3	12.7	12.2	10.7	11.7
Dicalcium phosphate anhydrous	0.50	0.50	1.00	3.00	0.50
Sodium chloride	4.50	4.40	3.20	4.40	3.70
L-Lysine HCl	4.30	3.80	5.30	4.15	4.40
L-Threonine	1.60	1.20	2.20	1.15	1.60
DL-Methionine	1.30	0.70	2.20	1.30	1.20
L-Tryptophan	0.20	0.10	0.20	0.20	0.10
L-Valine	0.00	0.00	0.30	0.00	0.00
Vitamin and trace mineral mixture^c^	1.20	1.20	1.20	1.20	1.20
Calculated/analysed composition^d^, % as fed or as specified					
Dry matter, analysed	88.00	87.70	88.30	87.90	87.90
Ash, analysed	3.90	3.60	4.00	4.10	3.90
NE, MJ/kg	10.03	10.03	10.03	9.61	10.45
SID Lys:NE, g/MJ	0.95	0.75	1.15	0.99	0.91
Crude Protein, analysed	13.40	11.60	16.20	14.50	14.30
Total Lys, analysed	1.05	0.88	1.31	1.08	1.02
Total Thr/Lys ratio, analysed	0.58	0.58	0.56	0.57	0.62
Total Met-Cys/Lys ratio, analysed	0.64	0.64	0.63	0.65	0.68
Total Trp/Lys ratio, analysed	0.14	0.15	0.15	0.14	0.14
Total Val/Lys ratio, analysed	0.60	0.65	0.65	0.67	0.69
Total Leu/Lys ratio, analysed	1.09	1.14	1.08	1.14	1.14
Total Ile/Lys ratio, analysed	0.54	0.55	0.57	0.58	0.60
Total His/Lys ratio, analysed	0.34	0.38	0.37	0.36	0.38
Total Phe/Lys ratio, analysed	0.61	0.64	0.65	0.69	0.69
Total Tyr/Lys ratio, analysed	0.25	0.25	0.30	0.32	0.30
Total Arg/Lys ratio, analysed	0.81	0.82	0.86	0.89	0.88
SID Lys	0.95	0.75	1.15	0.95	0.95
SID Thr/Lys ratio	0.65	0.65	0.65	0.65	0.65
SID Met-Cys/Lys ratio	0.59	0.59	0.59	0.59	0.59
SID Trp/Lys ratio	0.19	0.19	0.19	0.19	0.19
SID Val/Lys ratio	0.66	0.67	0.65	0.66	0.66
SID Leu/Lys ratio	1.16	1.20	1.11	1.16	1.15
SID Ile/Lys ratio	0.56	0.55	0.57	0.57	0.57
SID His/Lys ratio	0.36	0.36	0.34	0.35	0.36
Fat, analysed	2.79	2.74	3.78	2.21	4.19
Crude fiber, analysed	2.90	3.40	4.20	4.20	2.40
NDF	12.96	14.15	13.54	15.02	12.00
Calcium	0.75	0.75	0.80	0.77	0.72
Digestible phosphorus	0.22	0.22	0.22	0.25	0.22

^a^Diets were fed in finishing pigs during 10 days at 18 weeks of age

^b^C1 = Control; LP1 = Low Crude Protein; HP1 = High Crude Protein; LE1 = Low Net Energy; HE1 = High Net Energy

^c^Provided per each kg of feed: 60 mg Copper sulphate, 80 mg Ferrous sulphate monohydrate, 50 mg Manganese oxide, 100 mg Zinc oxide, 0.5 mg Potassium iodate, 0.4 mg Sodium selenite, 2 MIU Vitamin A, 0.5 MIU Vitamin D_3_, 40 MIU Vitamin E, 4 mg Vitamin K, 0.015 mg Vitamin B_12_, 2 mg Riboflavin, 12 mg Nicotinic acid, 10 mg Pantothenic acid, 2 mg Vitamin B_1_, 3 mg Vitamin B_6_

^d^NE = Net Energy; SID = Standardized Ileal Digestible; NDF = Neutral Detergent Fiber

The second study (**S2**) was conducted in growing pigs from 12 to 15 weeks of age (41.3 ± 2.60 to 62.1 ± 3.66 kg of BW). A total of 308 Danish Duroc × (Large White × Landrace) growing pigs born within one week were moved to the grower-finisher stage at 11 weeks of age and housed in balanced mixed sex pens. After one week of adaptation, pigs were weighed per pen (n = 28 pens; 11 pigs/pen) and assigned per pen based on BW to seven different dietary treatments at 12 weeks of age, following a complete randomized design. Pigs were followed for 20 days. Diets were formulated to obtain a control diet (**C2**; 10.03 MJ/kg of NE, 16.5% of CP, and 0.95% of SID Lys) which met or exceed the minimum nutrient requirements [26], and 6 modifications of this diet: low crude protein **(LP2)** by reducing CP to 14.0%; high crude protein **(HP2)** by increasing CP to 19.0%; low amino acid **(LA2)** by reducing AAs to 0.75% of SID Lys; high amino acid **(HA2)** by increasing AAs to 1.15% of SID Lys; low net energy **(LE2)** by reducing NE to 9.61 MJ/kg; and high net energy **(HE2)** by increasing NE to 10.45 MJ/kg. When the level of amino acids was modified all amino acids were adjusted following the ideal protein concept [26, 27]. Ingredient, calculated, and analysed nutrient diet composition is shown in Table 2. Pigs went back to the common management of the Teagasc Pig Research Facility after the 20-day trial period.

**Table 2:** Ingredient, calculated, and analysed nutrient composition on an as-fed basis of the seven dietary treatments in study 2^a^

	Diets^b^						
	C2	LP2	HP2	LA2	HA2	LE2	HE2
Ingredients, g/kg							
Maize	272.9	401.1	254.9	350.0	423.1	300.0	332.1
Barley	251.8	230.0	252.2	295.9	150.0	246.1	225.7
Wheat	218.1	150.0	150.0	150.0	150.0	150.0	150.0
Soybean meal 47.5	209.3	137.2	286.7	135.9	197.6	209.8	218.7
Soybean hulls	20.0	44.0	20.0	38.9	39.8	70.4	20.0
Vegetable oil	5.00	5.00	17.6	5.00	5.00	0.00	31.0
Calcium carbonate	10.6	14.0	10.5	13.6	12.3	11.3	10.6
Dicalcium phosphate anhydrous	0.50	0.50	0.50	0.50	0.70	0.50	0.50
Sodium chloride	4.80	5.00	4.30	4.50	5.20	4.90	4.50
L-Lysine HCl	3.00	5.20	0.80	2.60	6.00	2.90	2.90
L-Threonine	1.30	2.30	0.40	1.00	2.80	1.30	1.30
DL-Methionine	1.20	1.80	0.70	0.60	2.50	1.30	1.20
L-Tryptophan	0.10	0.50	0.00	0.10	0.60	0.10	0.10
L-Valine	0.00	0.90	0.00	0.00	1.30	0.00	0.00
L-Isoleucine	0.00	0.90	0.00	0.00	0.70	0.00	0.00
L-Leucine	0.00	0.00	0.00	0.00	0.60	0.00	0.00
L-Histidine	0.00	0.20	0.00	0.00	0.40	0.00	0.00
Vitamin and trace mineral mixture^c^	1.40	1.40	1.40	1.40	1.40	1.40	1.40
Calculated/analysed composition^d^, % as fed or as specified							
Dry matter, analysed	87.30	87.70	88.10	87.70	88.00	87.50	88.30
Ash, analysed	3.60	4.10	4.20	4.10	4.20	4.10	3.80
NE, MJ/kg	10.03	10.07	10.03	10.03	10.07	9.61	10.45
SID Lys:NE, g/MJ	0.95	0.94	0.95	0.75	1.14	0.99	0.91
Crude protein, analysed	15.4	15.1	17.7	13.7	16.6	16.2	16.3
Total Lys, analysed	1.02	1.12	1.08	0.86	1.32	1.07	1.02
Total Thr/Lys ratio, analysed	0.71	0.69	0.73	0.74	0.67	0.68	0.72
Total Met-Cys/Lys ratio, analysed	0.62	0.62	0.63	0.64	0.58	0.60	0.63
Total Trp/Lys ratio, analysed	0.19	0.15	0.18	0.09	0.15	0.16	0.17
Total Val/Lys ratio, analysed	0.75	0.68	0.87	0.76	0.67	0.74	0.75
Total Leu/Lys ratio, analysed	1.19	0.98	1.38	1.27	1.04	1.18	1.23
Total Ile/Lys ratio, analysed	0.63	0.56	0.76	0.64	0.57	0.63	0.64
Total His/Lys ratio, analysed	0.41	0.37	0.48	0.40	0.37	0.41	0.42
Total Phe/Lys ratio, analysed	0.70	0.55	0.82	0.73	0.57	0.64	0.69
Total Tyr/Lys ratio, analysed	0.29	0.23	0.28	0.23	0.27	0.25	0.25
Total Arg/Lys ratio, analysed	0.95	0.74	1.16	0.96	0.78	0.93	0.98
SID Lys	0.95	0.95	0.95	0.75	1.15	0.95	0.95
SID Thr/Lys ratio	0.65	0.65	0.65	0.65	0.65	0.65	0.65
SID Met-Cys/Lys ratio	0.59	0.59	0.59	0.59	0.59	0.59	0.59
SID Trp/Lys ratio	0.19	0.19	0.22	0.19	0.19	0.19	0.19
SID Val/Lys ratio	0.69	0.65	0.81	0.72	0.65	0.69	0.69
SID Leu/Lys ratio	1.14	0.98	1.33	1.23	0.99	1.15	1.16
SID Ile/Lys ratio	0.59	0.55	0.71	0.60	0.52	0.59	0.60
SID His/Lys ratio	0.37	0.32	0.43	0.38	0.32	0.36	0.37
Fat, analysed	2.28	2.75	3.91	2.64	2.89	2.40	5.00
Crude fiber, analysed	2.90	3.50	3.20	3.70	3.20	4.80	2.90
NDF	12.52	13.72	12.00	14.00	12.72	14.81	12.00
Calcium	0.70	0.80	0.70	0.80	0.77	0.75	0.70
Digestible phosphorus	0.22	0.22	0.22	0.22	0.22	0.22	0.22

^a^Diets were fed in growing pigs during 20 days at 12 weeks of age

^b^C2 = Control; LP2 = Low Crude Protein; HP2 = High Crude Protein; LA2 = Low Amino Acid; HA2 = High Amino Acid; LE2 = Low Net Energy; HE2 = High Net Energy

^c^Provided per each kg of feed: 60 mg Copper sulphate, 80 mg Ferrous sulphate monohydrate, 50 mg Manganese oxide, 100 mg Zinc oxide, 0.5 mg Potassium iodate, 0.4 mg Sodium selenite, 2 MIU Vitamin A, 0.5 MIU Vitamin D_3_, 40 MIU Vitamin E, 4 mg Vitamin K, 0.015 mg Vitamin B_12_, 2 mg Riboflavin, 12 mg Nicotinic acid, 10 mg Pantothenic acid, 2 mg Vitamin B_1_, 3 mg Vitamin B_6_

^d^NE = Net Energy; SID = Standardized Ileal Digestible; NDF = Neutral Detergent Fiber

### Body weight, feed intake, and feed efficiency traits

On both studies, S1 and S2, pigs were weighed per pen at the beginning and at the end of the trial period. Feed bags were used to feed the pigs. Overall feed intake was recorded per pen by weighing the feed bags before feeding the pigs and before weighing the pigs per pen (including the feed inside the feeder) at the end of the trial. Average daily gain **(ADG)**, average daily feed intake **(ADFI)** and feed conversion ratio **(FCR)** were calculated for the overall trial period. Feed conversion ratio was calculated as $\frac{\mathrm{kg of feed consumed}}{\mathrm{BW gain}}$.

### Feed analysis

Feed samples of each diet were collected per duplicate and analysed for dry matter, ash, CP, crude fibre, fat, and total AA profile at the Sciantec Analytical Services (Stockbridge Technology Centre, Cawood, Yorkshire, UK). Dry matter was measured by oven drying for 4 h at 103 °C [28]; ash was measured via combustion in a muffle furnace at 550 °C [29]; CP was determined as N × 6.25 based on the DUMAS method [30] using LECO FP-628 analyser (Leco Instruments Ltd., Stockport, UK); crude fibre was determined by a Fibertec semi-automatic system (Tecator, Hoganas, Sweden) using the gravimetry method [28]; fat was measured using Randall/Soxtec/Submersion method [31]; and total AA profile was determined based on ion exchange HPLC [32] using the Biochrom AA Analyser Sodium System (Biochrom Ltd., Cambridge, UK).

### Blood sample collection and blood serum analysis

Blood samples were collected via venepuncture of the external jugular vein (approximately 10 ml/pig) from 2 pigs/pen selected randomly at 10 days of the trial in S1 (20 weeks of age) and at 14 days of the trial in S2 (14 weeks of age). A total of 40 and 56 (n = 8 per treatment) blood samples were collected in S1 and S2 respectively. Blood samples were collected early in the morning in a non-fasting state as per commercial practice. Blood samples were kept immediately on ice at 4 °C after collection until serum was separated by centrifugation for 15 min at 2000 rcf. Blood serum samples were analysed the same day using the ABX Pentra 400 Clinical Chemistry benchtop analyser (HORIBA Medical, Irvine, California, USA) and ABX Pentra 400 re-agents (HORIBA ABX SAS, Montpellier, France) at the Teagasc Chemistry Lab in Fermoy, Co. Cork, Ireland. Selected blood serum metabolites (and techniques) were: albumin (bromocresol green dye-binding procedure), glucose (hexokinase method), triglycerides (enzymatic method), cholesterol (enzymatic photometric test), SUN (enzymatic UV test), total protein concentration (Biuret reaction), and creatinine (enzymatic method).

### Faecal sample collection and volatile fatty acid analysis

Faecal samples were collected using BioFreeze™ vials (Alimetrics Diagnostics Ltd, Espoo, Finland) from 2 pigs/pen selected randomly at 10 days of the trial in S1 (20 weeks of age) and at 14 days of the trial in S2 (14 weeks of age). Faecal samples were collected from pigs which defecated at the same moment of the sampling. A total of 40 and 56 (n = 8 per treatment) faecal samples were collected in S1 and S2 respectively. BioFreeze™ vials enable to collect the fresh samples and stop all biological activity at ambient temperature until the analysis. Faecal VFA analysis was conducted via gas chromatography using pivalic acid as an internal standard [33] at Alimetrics Diagnostics. The VFA profile included acetic, propionic, butyric, valeric, BCFA and total VFA.

### Data management and statistical analysis

Growth performance, blood serum and faecal VFA data analyses were carried out using SAS v9.4 (SAS Institute Inc., Cary, NC, USA). Plots were created using R v4.0.2 (R Foundation for Statistical Computing, Vienna, Austria). Pen was considered as the experimental unit for all performance, serum and faecal data analyses. Alpha level for determination of significance was 0.05 and trends were identified as alpha of 0.10. Data were tested for normality using the Shapiro–Wilk test and by examining the normal probability plot. Initial BW data were analysed using general linear models including treatment as fixed effect. Models for BW, ADG, ADFI, and FCR variables were analysed using general linear models including treatment diet as fixed effect and initial BW as a co-variable. For blood serum, models for albumin, glucose, triglycerides, cholesterol, SUN, creatinine, and total protein were analysed using general linear models including treatment diet as fixed effect. For faecal VFA, models for acetic, propionic, butyric, valeric, BCFA, and VFA were analysed using general linear models including treatment diet as fixed effect. Multiple means comparisons were done using Tukey–Kramer’s correction in all cases. Results for fixed effects are reported as least square means ± standard error mean.

Receiver Operating Characteristic **(ROC)** Curves analysis was used to identify biomarkers that discriminated between diets. Data were analysed using the *pROC* package [34] for R v4.0.2. Univariable ROC curves were calculated for SUN and BCFA comparing LP1 versus HP1, LE1 versus HE1, LP2 versus HP2, LE2 versus HE2, and LA2 versus HA2. The accuracy of the models was assessed by calculating the area under the curve **(AUC)**. Values of AUC were interpreted as non-accurate (AUC = 0.5), less accurate (0.5 < AUC ≤ 0.7), moderately accurate (0.7 < AUC ≤ 0.9), highly accurate (0.9 < AUC < 1) and perfect (AUC = 1) [35]. The AUC is significant when the confidence interval does not include 50%. Cut-off concentrations were calculated for each ROC curve and the corresponding sensitivities and specificities, and 95% confidence intervals were obtained [36].

## Results

### Body weight, feed intake, and feed efficiency traits

In S1, there were no differences on BW, ADG, ADFI, and FCR between dietary treatments (Table 3). In S2, pigs fed the LA2 diet were 3.5 kg lighter and gained 171.0 g/d less in average than those pigs fed with the C2, LP2, HP2, HA2, and HE2 diets at the end of the trial (*P* < 0.01; Table 4). Moreover, pigs fed the LP2 diet consumed 229.3 g/d more than HP2 pigs (*P* = 0.035) at the end of the trial. Finally, pigs fed the LA2 diet had higher FCR than pigs fed with the C2, HP2, HA2, LE2, and HE2 diets (*P* < 0.001); while HP2 pigs had lower FCR than those pigs fed with the C2, LP2, LA2, and LE2 dietary treatments (*P* < 0.001) at the end of the trial.

**Table 3:** Productive performance of finishing pigs grouped by dietary treatment in study 1^1^

	Dietary treatment^2^					SEM	*p*-Value
Traits	C1	LP1	HP1	LE1	HE1		
BW 128 days, kg	87.1	88.1	87.8	85.4	86.9	2.32	0.926
BW 138 days, kg	100.2	99.9	101.0	100.2	100.2	0.70	0.828
ADG, g	1315.0	1283.7	1399.1	1312.1	1317.7	71.49	0.825
ADFI, g	3024.2	3078.4	3110.0	3284.8	2977.7	128.86	0.531
FCR	2.33	2.39	2.23	2.48	2.27	0.08	0.228

^1^Body weight (BW), average daily gain (ADG), average daily feed intake (ADFI), feed conversion ratio (FCR) (SEM = Standard error mean [SEM]). Pigs were followed from 128 to 138 days of age (n = 4)

^2^Dietary treatments: C1 (Control; 10.03 MJ/kg of NE; 160.0 g of CP; 9.5 g of SID Lys per kg of feed), LP1 (Low Protein; 10.03 MJ/kg of NE; 132.0 g of CP; 7.5 g of SID Lys per kg of feed), HP1 (High Protein; 10.03 MJ/kg of NE; 188.0 g of CP; 11.5 g of SID Lys per kg of feed), LE1 (Low Energy; 9.61 MJ/kg of NE; 160.0 g of CP; 9.5 g of SID Lys per kg of feed), and HE1 (High Energy; 10.45 MJ/kg of NE; 160.0 g of CP; 9.5 g of SID Lys per kg of feed)

**Table 4:** Productive performance of growing pigs grouped by dietary treatment in study 2^1^

Traits	Dietary treatment^2^							SEM	*p*-Value
	C2	LP2	HP2	LA2	HA2	LE2	HE2		
BW 84 days, kg	41.1	41.7	41.3	41.5	41.1	41.2	41.3	1.49	1.000
BW 104 days, kg	62.6 ^a^	62.1 ^a^	62.7 ^a^	59.3 ^b^	63.2 ^a^	61.5 ^ab^	63.2 ^a^	0.57	0.001
ADG, g	1061.0 ^a^	1038.9 ^a^	1075.5 ^a^	900.9 ^b^	1090.1 ^a^	1011.5 ^ab^	1093.8 ^a^	28.90	0.002
ADFI, g	1932.3 ^ab^	1950.2 ^a^	1720.9 ^b^	1819.7 ^ab^	1933.2 ^ab^	1850.3 ^ab^	1878.4 ^ab^	46.76	0.030
FCR	1.84 ^b^	1.89 ^ab^	1.61 ^c^	2.03 ^a^	1.78 ^bc^	1.85 ^b^	1.73 ^bc^	0.04	< 0.001

^1^Body weight (BW), average daily gain (ADG), average daily feed intake (ADFI) and feed conversion ratio (FCR) (SEM = Standard error mean [SEM]). Pigs were followed from 84 to 104 days of age (n = 4)

^2^Dietary treatments: C2 (Control; 10.03 MJ/kg NE; 165.0 g of CP; 9.5 g of SID Lys per kg of feed), LP2 (Low Protein; 10.07 MJ/kg NE; 140.0 g of CP; 9.5 g of SID Lys per kg of feed), HP2 (High Protein; 10.03 MJ/kg NE; 190.0 g of CP; 9.5 g of SID Lys per kg of feed), LA2 (Low Amino Acid; 10.03 MJ/kg NE; 135.0 g of CP; 7.5 g of SID Lys per kg of feed), HA2 (High Amino Acid; 10.07 MJ/kg NE; 165.0 g of CP; 11.5 g of SID Lys per kg of feed), LE2 (Low Energy; 9.61 MJ/kg NE; 165.0 g of CP; 9.5 g of SID Lys per kg of feed), and HE2 (High Energy; 10.45 MJ/kg NE; 165.0 g of CP; 9.5 g of SID Lys per kg of feed)

^a^^−^^b^Within rows, significant differences between groups (*P* < 0.05)

### Blood serum metabolites

**Fig. 1:**
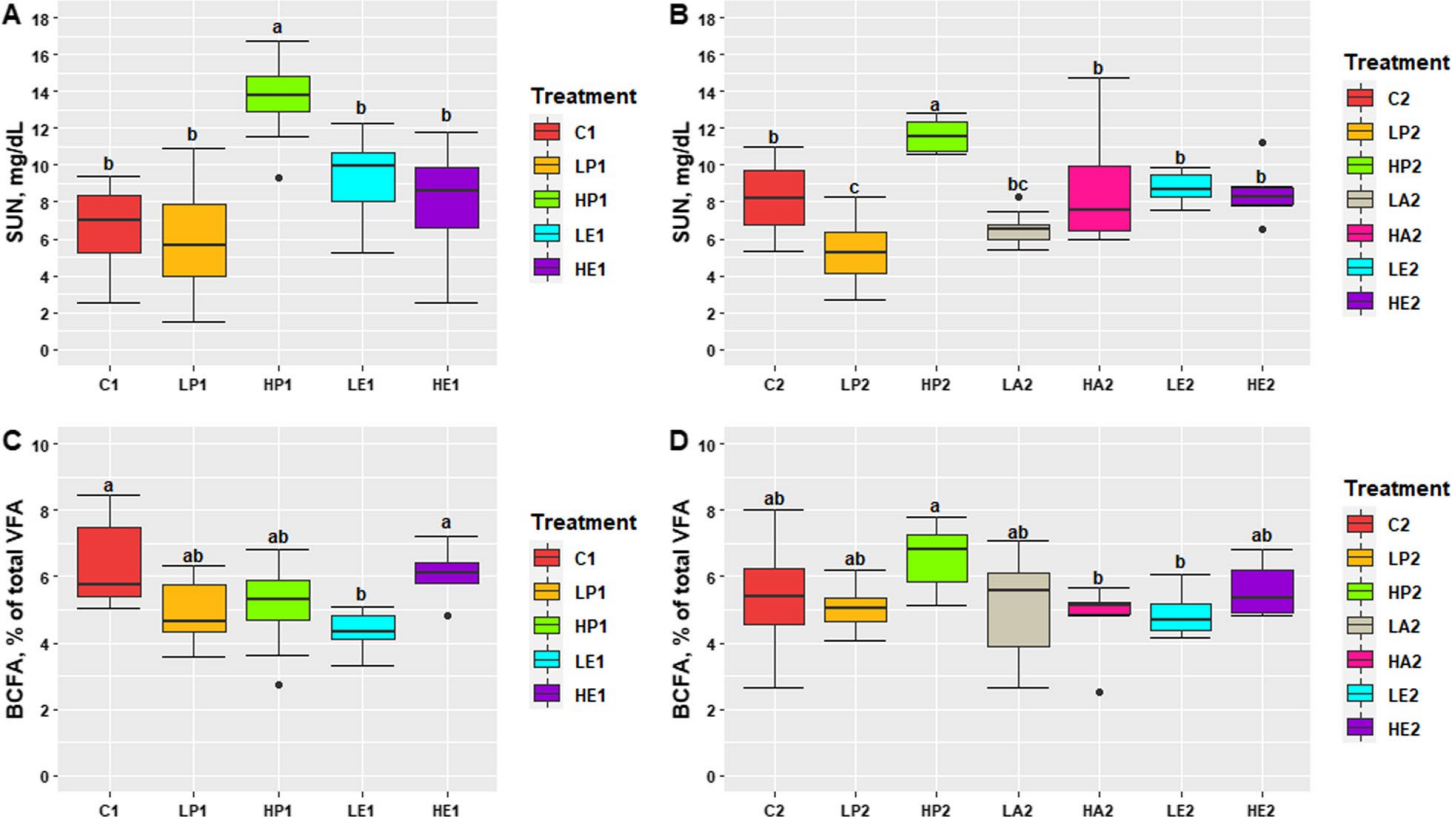
Serum urea nitrogen (SUN; mg/dL) and branched-chain fatty acids (BCFA; % of total VFA) levels grouped by dietary treatment (n = 4) from study 1 (**A**, **C**) and study 2 (**B**, **D**). Results are presented as Means ± Standard error mean. ^a, b^ Significant differences between treatments (*P* < 0.05). Blood and faecal samples were collected from 2 pigs/pen selected randomly at 20 weeks of age (study 1) and 14 weeks of age (study 2). Dietary treatments from study 1: C1 (Control; 10.03 MJ/kg of NE; 160.0 g of CP; 9.5 g of SID Lys per kg of feed), LP1 (Low Protein; 10.03 MJ/kg of NE; 132.0 g of CP; 7.5 g of SID Lys per kg of feed), HP1 (High Protein; 10.03 MJ/kg of NE; 188.0 g of CP; 11.5 g of SID Lys per kg of feed), LE1 (Low Energy; 9.61 MJ/kg of NE; 160.0 g of CP; 9.5 g of SID Lys per kg of feed), and HE1 (High Energy; 10.45 MJ/kg of NE; 160.0 g of CP; 9.5 g of SID Lys per kg of feed). Dietary treatments from study 2: C2 (Control; 10.03 MJ/kg NE; 165.0 g of CP; 9.5 g of SID Lys per kg of feed), LP2 (Low protein; 10.07 MJ/kg NE; 140.0 g of CP; 9.5 g of SID Lys per kg of feed), HP2 (High protein; 10.03 MJ/kg NE; 190.0 g of CP; 9.5 g of SID Lys per kg of feed), LA2 (Low amino acid; 10.03 MJ/kg NE; 135.0 g of CP; 7.5 g of SID Lys per kg of feed), HA2 (High amino acid; 10.07 MJ/kg NE; 165.0 g of CP; 11.5 g of SID Lys per kg of feed), LE2 (Low energy; 9.61 MJ/kg NE; 165.0 g of CP; 9.5 g of SID Lys per kg of feed), and HE2 (High energy; 10.45 MJ/kg NE; 165.0 g of CP; 9.5 g of SID Lys per kg of feed)

Blood metabolite profile for S1 and S2 are presented in Tables 5 and 6, respectively. In S1, albumin and glucose did not differ between dietary treatments, although HE1 pigs tended to have higher glucose concentration levels than LE1 pigs (*P* = 0.063). For creatinine and triglycerides, HE1 pigs had higher levels than LE1 pigs (*P* < 0.05) however, HE1 pigs had lower total protein levels than C1 pigs (*P* = 0.033). Furthermore, LP1 pigs had higher cholesterol concentration levels than LE1 pigs (*P* = 0.015) while HP1 pigs had higher SUN (13.63 ± 0.951 mg/dL) than the other dietary treatments (7.47 ± 0.951 mg/dL; *P* < 0.001; Fig. 1). In S2, glucose and creatinine did not differ between dietary treatments. For albumin, LA2 pigs had lower concentrations than pigs fed with HP2, HA2, LE2, and HE2 diets (*P* < 0.001). Also, LA2 pigs had lower total protein levels than HP2 pigs (*P* = 0.05). Pigs fed HP2 diet had higher SUN (11.60 ± 0.613 mg/dL) than the rest of the dietary treatments (7.65 ± 0.613 mg/dL; *P* < 0.001; Fig. 1). while LP2 pigs had lower SUN (5.2 ± 0.61 mg/dL) than those pigs fed with C2 (8.2 ± 0.61), HA2 (8.7 ± 0.61), LE2 (8.8 ± 0.61), HE2 (8.5 ± 0.61), and HP2 (*P* < 0.001). Finally, C2 pigs showed lower triglycerides levels than pigs fed with LE2 and HE2 dietary treatments (*P* < 0.01); however, HE2 pigs had higher cholesterol concentration levels than C2 and LE2 pigs (*P* < 0.05).

**Table 5:** Blood serum and faecal volatile fatty acid (VFA) profile from 40 finishing pigs grouped by dietary treatment (n = 4) in study 1^1^

Traits	Dietary treatment ^2^					SEM	*p*-Value
	C1	LP1	HP1	LE1	HE1		
Blood serum							
Albumin, g/L	36.54	37.08	38.04	36.04	36.53	0.880	0.565
Glucose, mmol/L	5.31	5.24	5.39	4.61	5.68	0.273	0.105
Triglycerides, mmol/L	0.38 ^ab^	0.35 ^ab^	0.37 ^ab^	0.28 ^b^	0.42 ^a^	0.030	0.023
Cholesterol, mmol/L	2.12 ^ab^	2.44 ^a^	2.27 ^ab^	2.03 ^b^	2.24 ^ab^	0.084	0.021
Creatinine, µmol/L	123.03 ^ab^	127.50 ^ab^	123.85 ^ab^	117.20 ^b^	133.81 ^a^	3.966	0.070
Total protein, g/L	67.36 ^a^	64.20 ^ab^	63.61 ^ab^	61.46 ^ab^	60.86 ^b^	1.509	0.035
Faecal VFA, % of total VFA							
Acetic	57.4 ^b^	61.6 ^a^	62.0 ^a^	60.6 ^ab^	58.5 ^ab^	1.01	0.012
Propionic	21.9	21.4	21.2	22.0	21.7	0.59	0.827
Butyric	11.4	9.8	9.3	10.9	11.0	0.55	0.061
Valeric	2.9 ^a^	2.3 ^ab^	2.4 ^ab^	2.1 ^b^	2.6 ^ab^	0.15	0.010
VFA, mmol/kg	193.0	158.2	166.9	170.5	154.7	14.98	0.451

^1^Blood and faecal samples were collected from 2 pigs/pen selected randomly at the end of the trial at 20 weeks of age (Means ± Standard error mean [SEM])

^2^Dietary treatments: C1 (Control; 10.03 MJ/kg of NE; 160.0 g of CP; 9.5 g of SID Lys per kg of feed), LP1 (Low Protein; 10.03 MJ/kg of NE; 132.0 g of CP; 7.5 g of SID Lys per kg of feed), HP1 (High Protein; 10.03 MJ/kg of NE; 188.0 g of CP; 11.5 g of SID Lys per kg of feed), LE1 (Low Energy; 9.61 MJ/kg of NE; 160.0 g of CP; 9.5 g of SID Lys per kg of feed), and HE1 (High Energy; 10.45 MJ/kg of NE; 160.0 g of CP; 9.5 g of SID Lys per kg of feed)

^a^^−^^b^Within rows, significant differences between groups (*P* < 0.05)

**Table 6:** Blood serum and faecal volatile fatty acid (VFA) profile from 56 growing pigs grouped by dietary treatment (n = 4) in study 2^1^

Traits	Dietary treatment ^2^							SEM	*p*-Value
	C2	LP2	HP2	LA2	HA2	LE2	HE2		
Blood serum									
Albumin, g/L	36.04^ab^	34.76^ab^	38.05^a^	32.10^b^	39.08^a^	37.25^a^	37.19^a^	1.083	< 0.001
Glucose, mmol/L	5.37	5.83	5.72	5.94	6.05	5.69	5.57	0.253	0.559
Triglycerides, mmol/L	0.33^b^	0.55^ab^	0.44^ab^	0.57^ab^	0.47^ab^	0.67^a^	0.61^a^	0.059	0.004
Cholesterol, mmol/L	2.34^b^	2.61^ab^	2.45^ab^	2.52^ab^	2.39^ab^	2.35^b^	2.80^a^	0.107	0.046
Creatinine, µmol/L	111.01	100.29	117.56	113.89	112.97	113.00	107.34	4.422	0.168
Total protein, g/L	61.98^ab^	64.60^ab^	65.95^a^	59.98^b^	63.49^ab^	61.64^ab^	63.44^ab^	1.391	0.077
Faecal VFA, % of total VFA									
Acetic	55.99	55.22	54.64	54.66	56.84	56.37	56.01	1.192	0.798
Propionic	21.15^b^	23.18^ab^	21.40^b^	24.53^a^	21.19^b^	21.57^b^	21.51^b^	0.567	< 0.001
Butyric	13.53	12.57	13.25	11.81	13.42	13.04	13.42	0.710	0.608
Valeric	4.08	3.96	4.09	3.99	3.72	4.18	3.49	0.367	0.844
VFA, mmol/kg	93.78	88.44	91.55	86.58	95.75	99.86	88.38	6.281	0.754

^1^Blood and faecal samples were collected from 2 pigs/pen selected randomly at day 14 of the trial at 14 weeks of age (Means ± Standard error mean [SEM])

^2^Dietary treatments: C2 (Control; 10.03 MJ/kg NE; 165.0 g of CP; 9.5 g of SID Lys per kg of feed), LP2 (Low protein; 10.07 MJ/kg NE; 140.0 g of CP; 9.5 g of SID Lys per kg of feed), HP2 (High protein; 10.03 MJ/kg NE; 190.0 g of CP; 9.5 g of SID Lys per kg of feed), LA2 (Low amino acid; 10.03 MJ/kg NE; 135.0 g of CP; 7.5 g of SID Lys per kg of feed), HA2 (High amino acid; 10.07 MJ/kg NE; 165.0 g of CP; 11.5 g of SID Lys per kg of feed), LE2 (Low energy; 9.61 MJ/kg NE; 165.0 g of CP; 9.5 g of SID Lys per kg of feed), and HE2 (High energy; 10.45 MJ/kg NE; 165.0 g of CP; 9.5 g of SID Lys per kg of feed)

^a^^−^^b^Within rows, significant differences between groups (*P* < 0.05)

### Volatile fatty acids profile

Total VFA and VFA profiles for S1 and S2 are presented in Tables 5 and 6, respectively. In S1, total VFA (mmol/kg) did not differ between dietary treatments. Pigs fed C1 had lower percentage of acetic than LP1 and HP1 pigs (*P* < 0.05) but higher percentage of valeric than LE1 pigs (*P* = 0.010). Pigs fed LE1 showed lower percentage of BCFA (4.4 ± 0.38) of total VFA than C1 and HE1 pigs (6.4 ± 0.38; 6.1 ± 0.38, respectively; P < 0.01; Fig. 1). In S2, total VFA (mmol/kg) did not differ between dietary treatments (*P* > 0.05). Moreover, acetic, butyric, and valeric (as % of total VFA) did not differ between dietary treatments (*P* > 0.05). Nevertheless, LA2 pigs had higher percentage of propionic of total VFA than those pigs fed with C2, HP2, HA2, LE2, and HE2 dietary treatments (*P* < 0.001). Finally, HP2 pigs had higher percentage of BCFA (6.61 ± 0.408) of total VFA than HA2 and LE2 pigs (4.84 ± 0.408; 4.84 ± 0.408, respectively; *P* < 0.05; Fig. 1); and HP2 pigs tended to have higher percentage of BCFA of total VFA than LP2 and LA2 pigs (5.06 ± 0.408; 5.01 ± 0.408, respectively; *P* < 0.10; Fig. 1).

### ROC curve analysis

**Fig. 2:**
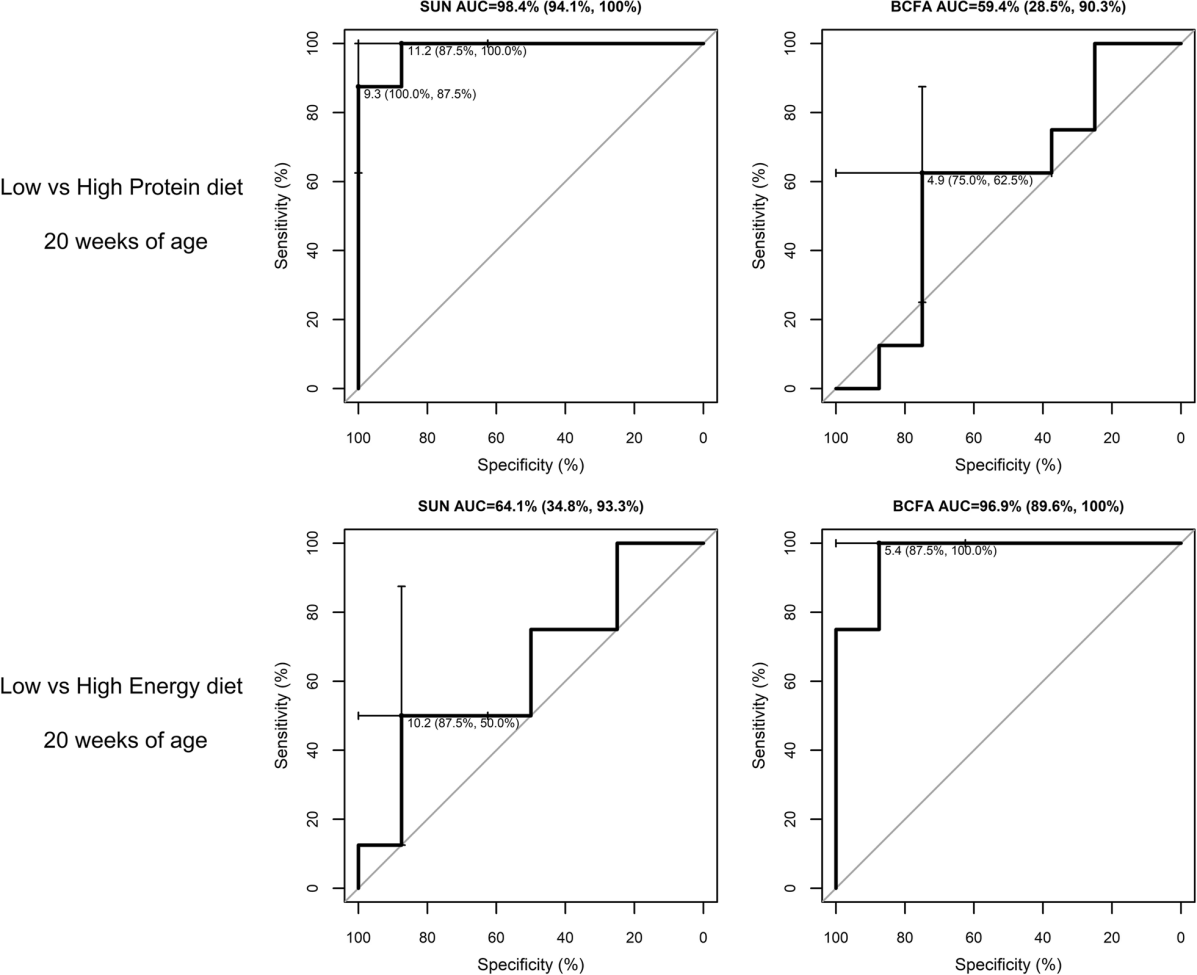
Receiver operating characteristic (ROC) curves for serum urea nitrogen (SUN) and branched-chain fatty acids (BCFA) biomarkers used to differentiate low versus high protein and energy diets in finishing pigs at 20 weeks of age (Study 1). Dietary treatments from study 1: low Protein (10.03 MJ/kg of NE; 132.0 g of CP; 7.5 g of SID Lys per kg of feed), High Protein (10.03 MJ/kg of NE; 188.0 g of CP; 11.5 g of SID Lys per kg of feed), Low Energy (9.61 MJ/kg of NE; 160.0 g of CP; 9.5 g of SID Lys per kg of feed), and High Energy (10.45 MJ/kg of NE; 160.0 g of CP; 9.5 g of SID Lys per kg of feed). Headings include the area under the curve (AUC) and the 95% confidence interval (CI). The AUC is significant when the CI does not include 50%. Within each graph, the optimal cut-off concentration and the corresponding specificity and sensitivity (parenthesis) are shown

**Fig. 3:**
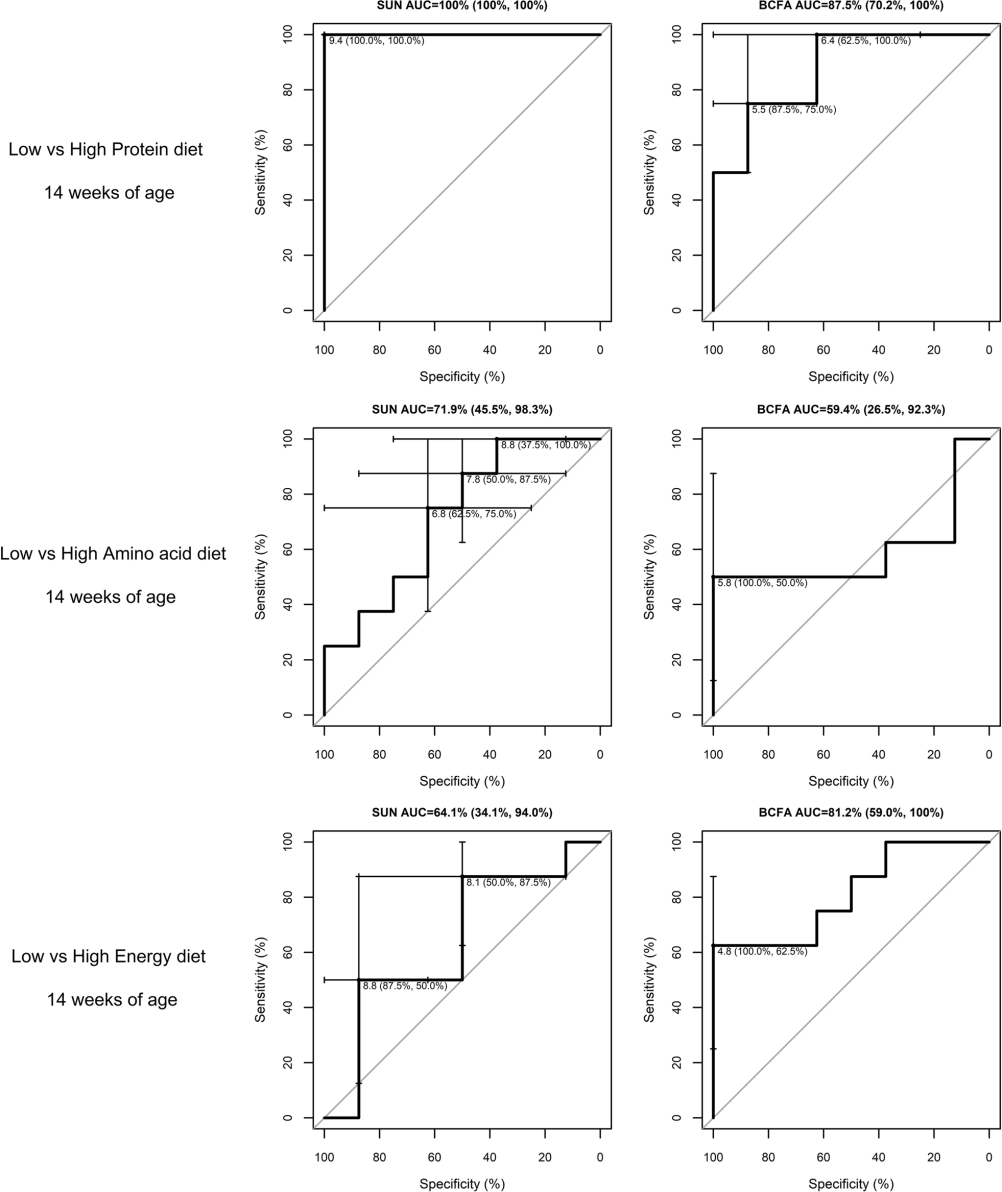
Receiver operating characteristic (ROC) curves for serum urea nitrogen (SUN) and branched-chain fatty acids (BCFA) biomarkers used to differentiate low versus high protein, amino acids, and energy diets in growing pigs at 14 weeks of age (Study 2). Dietary treatments from study 2: Low protein (10.07 MJ/kg NE; 140.0 g of CP; 9.5 g of SID Lys per kg of feed), High protein (10.03 MJ/kg NE; 190.0 g of CP; 9.5 g of SID Lys per kg of feed), Low amino acid (10.03 MJ/kg NE; 135.0 g of CP; 7.5 g of SID Lys per kg of feed), High amino acid (10.07 MJ/kg NE; 165.0 g of CP; 11.5 g of SID Lys per kg of feed), Low energy (9.61 MJ/kg NE; 165.0 g of CP; 9.5 g of SID Lys per kg of feed), and High energy (10.45 MJ/kg NE; 165.0 g of CP; 9.5 g of SID Lys per kg of feed). Headings include the area under the curve (AUC) and the 95% confidence interval (CI). The AUC is significant when the CI does not include 50%. Within each graph, the optimal cut-off concentration and the corresponding specificity and sensitivity (parenthesis) are shown

The ROC curve analysis for S1 is presented in Fig. 2. The AUC for SUN was of 98.4% with two optimal cut-offs of 9.4 mg/dL (100% sensitivity, 87.5% specificity) and 11.2 mg/dL (87.5% sensitivity, 100% specificity) that could serve to differentiate the LP1 versus HP1 dietary treatments. Branched-chain fatty acids showed a high accuracy (AUC = 96.9%) to differentiate the LE1 versus HE1 dietary treatments. The ROC curve analysis for S2 is presented in Fig. 3. The AUC for SUN was 100% with an optimal cut-off of 9.4 mg/dL to differentiate LP2 versus HP2 dietary treatments. Branched-chain fatty acids showed a moderate-high accuracy to differentiate LP2 versus HP2, and LE2 versus HE2 dietary treatments (AUC = 87.5% and 81.2%, respectively).

## Discussion

Although these trials were not designed to study differences in productive performance due to the short period of study and low sample size, productive performance was monitored and there were some interesting findings. In S1, there were no differences in productive performance among diets. Thus, for S2, the authors decided to use younger pigs which are more likely to be affected by reductions in dietary AA levels similar to those used in S1 (from 1.15 to 0.75 g SID lysine per MJ). Pigs fed LA2 diet were the lightest and had the worst efficiency of all the diets in S2, probably because the levels of AAs were not enough to meet the requirements of the pigs [26]. Pigs in S2 were also followed for a slightly longer period of time which may have allowed them to show differences in productive performance. It is interesting to note that productive performance of pigs fed LP2 diet was not affected compared to HP2. This diet was supplemented with AAs and both LP2 and HP2 had the same level of SID lysine (9.5 g per MJ). Thus, LP2 would achieve a lower risk of abnormal protein fermentations and lower emissions than HP2 with no negative effects in performance.

Serum metabolites have been used in pigs for research purposes but the literature on their use for clinical purposes is scarce and reference values are needed considering different factors such as age, breed, sex, diet, and methods of sample collection and analysis [37]. In this study, the authors aimed to use serum metabolites as biomarkers to discriminate between diets differing on CP, AA, and NE levels with the final intention to use these biomarkers in daily practice as indicator of suboptimal diets. In S1, SUN was the clearest indicator of differences between diets with high and low CP levels, which were discriminated in the ROC curve analysis with an AUC close to 1. The principal end product of protein catabolism is SUN [38], thus it makes sense that SUN increases when the diet has an excess of CP that cannot be used by the animal due to CP excess or AA imbalances. Diets LP1 and HP1 differed in both factors, the level of CP and the level of AAs. To separate the effect of these 2 factors, in S2, LP2 and HP2 were formulated to have different levels of CP but the same levels of AAs, and LA2 and HA2 were formulated to differ on AA levels without an excess on protein. As expected, diet HP2, with an excess of CP, resulted in an increased SUN level compared to LP2. However, diet HA2 compared to LA2 did not induce the same increase in SUN that HP2 compared to LP2, despite having a similar increase in CP. The increase in CP in diet HA2 could be used by the pig for growth because it was achieved by increasing AAs levels according to ideal protein profile. Overall, SUN may be a useful indicator of protein efficiency at farm level when pigs are fed suboptimal dietary CP diets not balanced in AAs. The use of SUN is also an advantage because of its short time to achieve a constant concentration in blood after changing the diet [20]. Further research is needed to fully understand the applicability of SUN in a cohort of commercial farms. Since there is no control diets at farm level, an empirical approach should be conducted within a farm and between farms to establish SUN standard intervals based on regressions and considering factors such as diets, feed form, age or BW, among others, when relevant. The infrastructure required for such analysis would be similar to those offered currently for small animal medicine where SUN and other biomarkers are used regularly.

Total protein and albumin are also involved in protein metabolism and were studied as interesting biomarkers. There were no differences in S1 in total protein and albumin between diets. The latter is in agreement with Regmi et al. [17], who did not observe differences in serum total protein concentration in finishing pigs, of similar age to those in S1, fed insufficient (0.32%), adequate (0.60%) or excessive (0.87%) SID lysine diets during 4 weeks, and only observed reduced plasma albumin concentration in finishing pigs when fed the 0.32% SID lysine diet which is far below the SID lysine levels of the dietary treatments of the present study. Nevertheless, in S2, pigs fed higher amounts of CP showed an increase in serum total protein and albumin levels. These findings are in accordance with some of the previous literature [16, 18]. Thus, the age of the pig may affect serum total protein and albumin levels and finishing pigs may be able to show a homeostatic control besides the dietary CP content. Moreover, finishing pigs have already reached the maximum protein deposition [39–41] and their metabolism may not be focused on protein turn-over, contrary to early stages of the grower-finisher period. Albumin was a good biomarker to differentiate between LA2 and HA2 in S2, but it was not as consistent as SUN. Further research is needed to explore its use in multivariable biomarkers in combination with SUN.

Serum creatinine is also related to protein metabolism because is the product from muscle metabolism [37] and has a positive correlation with total and striated muscle [42]. In the current studies, creatinine did not show any clear patterns and may not be as good as SUN as a biomarker.

Concerning energy, serum glucose did not show clear patterns between dietary treatments either which agrees with previous literature [15–17] and shows a good homoeostatic control of serum glucose concentration by either growing and finishing pigs in commercial conditions fed ad libitum. Triglycerides and cholesterol are both metabolites involved in lipid metabolism [37]. They did not show any consistent pattern despite showing some differences between diets differening in NE levels. The inconsistency in the results may be related to the age of the animals. The hypercholesterolemic effect observed in S2 pigs but not so clear in S1 is in agreement with previous literature [16–18], although the exact mecanishim of this effect is not clear yet. Further research should be carried out in order to further define these results.

No differences in total VFAs concentrations were observed between the dietary treatments in any of the two trials, and none of the individual VFAs showed difference worth discussing except for BCFA. The authors hypothesised that BCFA would show CP excess in the diets based on the previous literature [23, 25]. However, the pattern differed between trials. Growing pigs in S2 fed HP2 diet showed a higher percentage of BCFA than pigs fed the HA2 and LE2 diets. Moreover, HP2 pigs had numerically greatly amounts of BCFA compared to those pigs fed the LP2 and LA2 diets. ROC curve analysis showed that BCFA has a moderate-high accuracy to differentiate LP2 and HP2 diets in growing pigs. These findings agree with previous literature that reported an increased production of BCFA in manure in grower-finisher pigs fed high CP diets [23, 43]. The low production of BCFA in pigs fed the HA2 diet compared to those fed the HP2 diet might be related to a fast absorption rate of free AA and a lower level of CP available for fermentation which agrees with the pattern observed for SUN. In S1, finishing pigs fed the HP1 diet did not show higher percentage of BCFA than any other dietary treatment. This absence of differences in BCFA might be explained by the fact that older pigs have a more developed gastrointestinal tract with a high fermentation capacity that makes it difficult to observe differences between the dietary treatments at faecal level. The differences may exist in cecum or proximal colon but are not present in faeces. On the other hand, a higher percentage of BCFA between pigs was found in pigs fed diet HE1 when compared to LE1. This difference could be related to the added fibre in the LE1 diet. In this line, a recent study reported that the increased body weight and age of the pigs resulted in an improved digestibility of dietary fibre fractions [44], which will influence the VFA profile as it is positively correlated with the apparent total tract digestibility of insoluble dietary fibre and cellulose [45]. Therefore, the fermentation of soybean hulls could have produced a shift in the VFA profile reducing the BCFA production by the microbial population. Although BCFA did not show the same consistency as SUN, more research is warranted. The deamination of branched AAs may also cause a shift in the microbiome population to increase production of BCFA [25] which may be a more sensitive biomarker.

## Conclusion

This study aimed to assess the feasibility of blood serum metabolite and faecal VFA profiles as potential biomarkers to identify differences in the dietary levels of energy, protein, and amino acids in growing and finishing pigs at farm level when fed suboptimal diets. Out of all the blood serum metabolites studied, SUN seems to be the best indicator to assess protein efficiency at farm level showing high accuracy to detect an excess of crude protein with an inadequate amino acid profile in growing and finishing pig diets. Regarding the faecal VFA profile, BCFAs could be a potential indicator for high CP diets but may be affected by the age of the pig. Further studies at commercial scale are needed to fully understand the applicability of SUN in a cohort of commercial farms. The use of other biomarkers as part of a multivariable indicator should also be considered.

## Data Availability

The datasets used and analysed during the current study are available from the corresponding author on reasonable request.

## Abbreviations

AA: Amino Acids
ADFI: Average Daily Feed Intake
ADG: Average Daily Gain
BCFA: Branched-Chain Fatty Acids
BW: Body Weight
C1: Control Study 1
C2: Control Study 2
CP: Crude Protein
FCR: Feed Conversion Ratio
HA2: High Amino Acids Study 2
HE1: High Net Energy Study 1
HE2: High Net Energy Study 2
HP1: High Crude Protein Study 1
HP2: High Crude Protein Study 2
LA2: Low Amino Acids Study 2
LE1: Low Net Energy Study 1
LE2: Low Net Energy Study 2
LP1: Low Crude Protein Study 1
LP2: Low Crude Protein Study 2
Lys: Lysine
NE: Net Energy
S1: Study 1
S2: Study 2
SID: Standardized Ileal Digestible
SUN: Serum Urea Nitrogen
VFA: Volatile Fatty Acids
